# Tubular Peroxiredoxin 3 as a Predictor of Renal Recovery from Acute Tubular Necrosis in Patients with Chronic Kidney Disease

**DOI:** 10.1038/srep43589

**Published:** 2017-02-27

**Authors:** Chia-Lin Wu, Tzu-Cheng Su, Chia-Chu Chang, Chew-Teng Kor, Chung-Ho Chang, Tao-Hsiang Yang, Ping-Fang Chiu, Der-Cherng Tarng

**Affiliations:** 1 Division of Nephrology, Department of Internal Medicine, Changhua Christian Hospital, Changhua, Taiwan; 2 Institute of Clinical Medicine, National Yang-Ming University, Taipei, Taiwan; 3 School of Medicine, Chung-Shan Medical University, Taichung, Taiwan; 4 Internal Medicine Research Center, Changhua Christian Hospital, Changhua, Taiwan; 5 Department of Pathology, Changhua Christian Hospital, Taipei, Taiwan; 6 Environmental and Precision Medicine Laboratory, Changhua Christian Hospital, Changhua, Taiwan; 7 Institute of Cellular and System Medicine, National Health Research Institutes, Miaoli County, Taiwan; 8 Division of Nephrology, Department of Medicine, Taipei Veterans General Hospital, Taipei, Taiwan; 9 Department and Institute of Physiology, National Yang-Ming University, Taipei, Taiwan

## Abstract

Peroxiredoxin 3 (PRX3) is a mitochondrial antioxidant that regulates apoptosis in various cancers. However, whether tubular PRX3 predicts recovery of renal function following acute kidney injury (AKI) remains unknown. This retrospective cohort study included 54 hospitalized patients who had AKI with biopsy-proven acute tubular necrosis (ATN). The study endpoint was renal function recovery within 6 months. Of the 54 enrolled patients, 25 (46.3%) had pre-existing chronic kidney disease (CKD) and 33 (61%) recovered renal function. Tubular PRX3 expression was higher in patients with ATN than in those without renal function recovery. The level of tubular but not glomerular PRX3 expression predicted renal function recovery from AKI (AUROC = 0.76). In multivariate Cox regression analysis, high PRX3 expression was independently associated with a higher probability of renal function recovery (adjusted hazard ratio = 8.99; 95% CI 1.13–71.52, *P* = 0.04). Furthermore, the discriminative ability of the clinical model for AKI recovery was improved by adding tubular PRX3. High tubular PRX3 expression was associated with a higher probability of renal function recovery from ATN. Therefore, tubular PRX3 in combination with conventional predictors can further improve recovery prediction and may help with risk stratification in AKI patients with pre-existing CKD.

Acute kidney injury (AKI), one of the most serious health complications in communities or hospitals, is associated with high morbidity and mortality rates^1^,^2^. Tubular injury plays an important role in subsequent chronic kidney disease (CKD) following AKI, including vascular rarefaction, fibrosis, and glomerulosclerosis^3^. Once CKD has developed, renal function may gradually decline, and progression to end-stage renal disease (ESRD) is usually inevitable. Immediate treatment of AKI is required not only to reduce mortality from AKI but also subsequent CKD and ESRD, which are major global public health problems.

Oxidative stress is an important pathogenic factor of AKI, including ischemia-reperfusion injury-induced, sepsis-induced, and nephrotoxin-induced AKI^4^,^5^,^6^,^7^. Antioxidant defense systems involving proteins such as nuclear factor-erythroid 2 p45-related factor 2 (Nrf2), glutathione, and heme oxygenase were reported to be altered and have a protective role in AKI^7^,^8^. In addition, previous studies using experimental models of AKI have suggested that antioxidants, e.g., *N*-acetylcysteine, vitamin E, mitoquinone mesylate, or resveratrol, may ameliorate renal injury^9^. However, there is still a lack of clinically proven treatments for AKI, except for renal replacement therapy.

Peroxiredoxins (PRXs) are one of the major antioxidants in mammalian cells. PRXs are sensitive to oxidation by H_2_O_2_ and function as scavengers of intracellular H_2_O_2_^10^. There are six isoforms in mammalian cells (PRX1–6), and they have specific subcellular locations^11^. As the third member of the PRX family, PRX3 is mainly localized in the mitochondria^12^. Oberley *et al*. reported that PRX3 is abundant in the proximal and distal tubular cells of rats^13^. PRX3 regulates mitochondrial H_2_O_2_ and controls the mitochondrial apoptotic signaling pathway in HeLa and cancer cells^14^,^15^. Moreover, overexpression of PRX3 protects the heart from left ventricular remodeling and heart failure after myocardial infarction^16^. Although growing evidence suggests that PRX3 may respond to AKI in experimental animal models, the role of PRX3 in patients with AKI remains unclear^17^,^18^.

Little is known about the role of PRX3 in renal tubular injury. We hypothesized that PRX3 expression in the renal tubules may increase in response to AKI and be a prognostic factor for AKI. Therefore, in this study, we investigated the expression levels of PRX3 in the kidneys during AKI and examined the association between renal outcome and PRX3 expression after AKI.

## Results

### Demographic and Clinical Characteristics of Patients

We enrolled 68 patients with biopsy-proven acute tubular necrosis (ATN) in this retrospective cohort study. A total of 14 patients were excluded from this study because there was insufficient renal tissue for immunohistochemical staining (n = 12) or because they were kidney transplant recipients (n = 2). Twenty-five out of 54 (46.3%) patients had pre-existing CKD (baseline estimated glomerular filtration rate [eGFR] < 60 ml/min/1.73 m^2^). None of the patients started dialysis therapy at the time of kidney biopsy. The median follow-up was 1.3 (interquartile range, 0.4–8.2) months. During the follow-up period, 33 patients recovered kidney function, including 15 complete recoveries and 18 partial recoveries. Most complete recoveries (n = 13; 86.7%) occurred within the first 6 months after AKI. Among the 21 patients without recovery of renal function, 12 (57.1%) patients reached ESRD and required renal replacement therapy during the follow-up period.

Table 1 shows the demographic, clinical, and laboratory data of patients with recovery (recovery group, n = 33) and without recovery (non-recovery group, n = 21) of renal function. Patients of both groups were similar with respect to age, gender distribution, presence of heart failure, severity of AKI, levels of serum components (albumin, cholesterol, triglyceride, sodium, potassium, and phosphorus), tubular injury and interstitial inflammation scores, percentage of interstitial fibrosis, use of angiotensin-converting-enzyme inhibitors or angiotensin-II receptor blockers, and immunosuppressive treatment. Nevertheless, patients with recovery of renal function had lower instances of diabetes mellitus, hypertension, diabetic nephropathy, and glomerulonephritis and higher instances of normal glomeruli and tubulointerstitial disease. In addition, they had lower baseline serum creatinine, urinary protein-to-creatinine ratio, and percentage of tubular atrophy in the renal interstitium and higher eGFR and hemoglobin levels (all *P* < 0.05).

### Differential Expression of PRX3 in the Renal Tubules between the Recovery and Non-Recovery Groups

**Figure 1:**
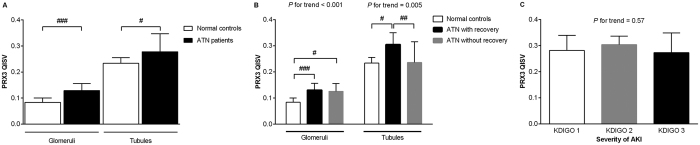
Peroxiredoxin 3 (PRX3) expression in the kidneys. (**A**) PRX3 was up-regulated in both glomeruli and tubules in patients with acute tubular necrosis (ATN) compared with normal controls. (**B**) Among patients with ATN, there was no difference in glomerular PRX3 quantitative immunohistochemical staining value (QISV) between patients with and without kidney function recovery. However, the tubular PRX3 QISV of recovered patients was higher compared with that of normal controls and non-recovered patients. (**C**) Tubular PRX3 QISV was not related to the severity of acute kidney injury (**C**). ^#^*P* < 0.05; ^##^*P* < 0.01; ^###^*P* < 0.001. KDIGO, Kidney Disease: Improving Global Outcomes.

The expression levels of renal PRX3 were higher in patients with ATN than in normal controls (Fig. 1A). Following the recovery of renal function from ATN, PRX3 expression was higher in the renal tubules of recovered patients than in those of non-recovered patients during the follow-up period (Fig. 1B). However, PRX3 expression in the renal tubules did not significantly differ among different AKI severity groups (Fig. 1C).

### Relationship of Tubular PRX3 Expression with Baseline eGFR, Urinary Protein Excretion, and Histopathology of AKI

**Figure 2:**
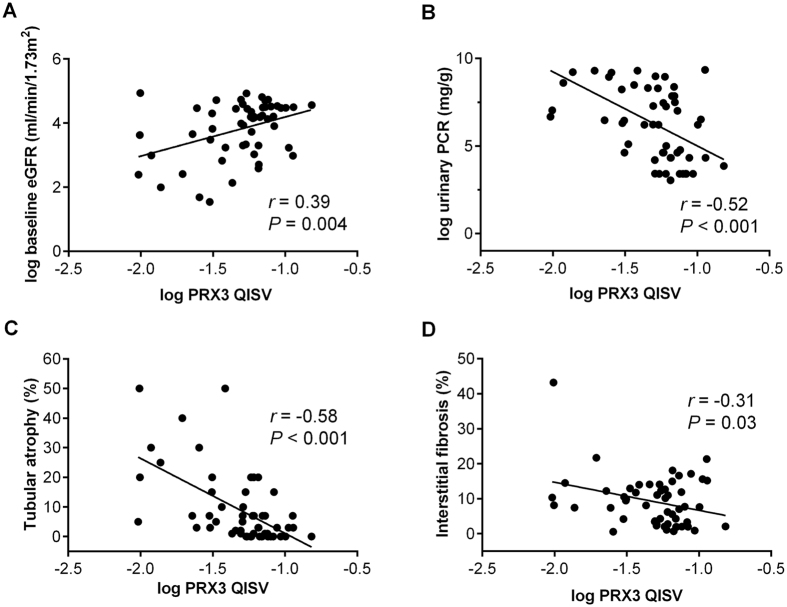
Relationship of tubular PRX3 expression with (**A**) baseline eGFR, (**B**) proteinuria, (**C**) tubular atrophy, and (**D**) interstitial fibrosis. eGFR, estimated glomerular filtration rate; PCR, protein-to-creatinine ratio; PRX3, peroxiredoxin 3; QISV, quantitative immunohistochemical staining value.

Correlation analyses showed that the expression level of tubular PRX3 was positively correlated with baseline eGFR (*r* = 0.39, *P* = 0.004; Fig. 2A) but negatively correlated with urinary protein-to-creatinine ratio (*r* = −0.52, *P* < 0.001; Fig. 2B), tubular atrophy (*r* = −0.58, *P* < 0.001 Fig. 2C), and interstitial fibrosis (*r* = −0.31, *P* = 0.03; Fig. 2D).

### Association of Tubular PRX3 Expression with Recovery of Renal Function

**Figure 3:**
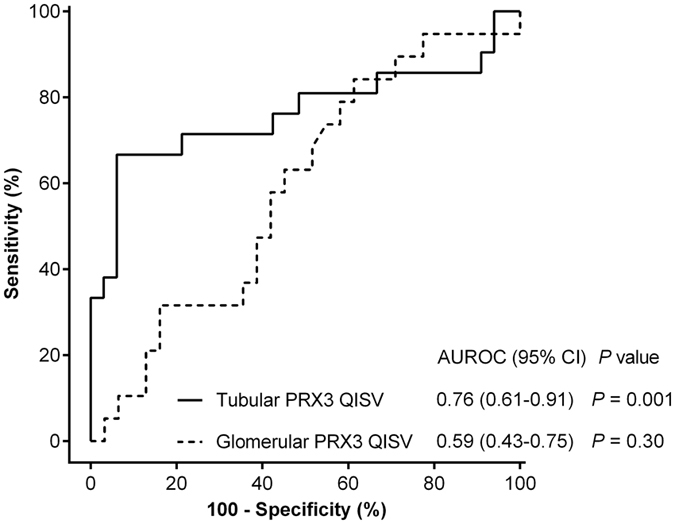
Tubular (solid line) and glomerular (dashed line) PRX3 as biomarkers in ROC analysis for predicting renal function recovery within 6 months following AKI. AUROC, area under the ROC curve; CI, confidence interval; eGFR, estimated glomerular filtration rate; PRX3, peroxiredoxin 3; QISV, quantitative immunohistochemical staining value; ROC, receiver operating characteristic.

**Figure 4:**
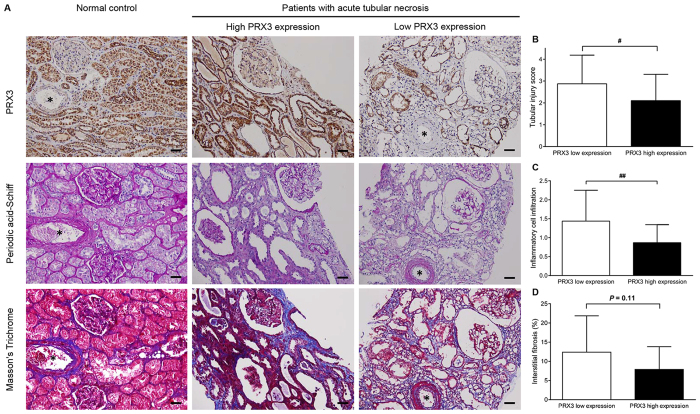
Representative images of immunohistochemical staining of PRX3, periodic acid-Schiff and Masson’s trichrome staining of kidney tissues from patients with acute tubular necrosis (ATN) and normal controls. (**A**) Asterisks indicate the blood vessels. (**B**) Tubular injury score, (**C**) inflammatory cell infiltration, and (**D**) the percentage of interstitial fibrosis were assessed by computer-assisted quantitative methods. Patients were stratified into high and low expression groups by the cutoff value of 0.2492 for tubular PRX3 QISV based on the ROC curve analysis. Data are expressed as mean ± SD. ^#^*P* < 0.05; ^##^*P* < 0.01. Scale bars, 50 μm. PRX3, peroxiredoxin 3; QISV, quantitative immunohistochemical staining value; ROC, receiver operating characteristic; SD, standard deviation.

In comparison with glomerular PRX3, tubular PRX3 exhibited a high accuracy in discriminating recovered patients from non-recovered patients, with an area under the receiver operating characteristic curve (AUROC) of 0.76 (95% CI 0.61–0.91, *P* = 0.001) (Fig. 3). Tubular PRX3 provided higher discrimination in predicting recovery from AKI than glomerular PRX3 (the C-index was 0.67 for tubular PRX3 and 0.56 for glomerular PRX3, Supplementary Table S1). The optimal cutoff value of tubular PRX3 quantitative immunohistochemical staining value (QISV) for predicting renal recovery based on the Youden’s index of receiver operating characteristic (ROC) analysis was 0.2492. According to the optimal cutoff value, the patients were stratified into two groups: those with high (n = 38) and low (n = 16) expression of tubular PRX3. The low PRX3 expression group had significantly higher scores for tubular injury and inflammatory cell infiltration than the high PRX3 expression group (Fig. 4). Kaplan-Meier curves revealed a significant increase in renal recovery in the high PRX3 expression group compared with the low PRX3 expression group (*P* < 0.001, see Supplementary Fig. S1). In age- and sex-adjusted Cox proportional hazards regression analysis (model 1; Table 2), high tubular PRX3 expression, baseline eGFR, urinary protein-to-creatinine ratio, and hemoglobin levels were significantly associated with kidney function recovery within 6 months after AKI (all *P* < 0.05). In multivariate Cox regression analysis (model 2 and 3, Table 2), high tubular PRX3 expression and hemoglobin levels remained independently associated with a higher probability of renal function recovery, with adjusted hazard ratios of 8.99 (95% CI 1.13–71.52, *P* = 0.04) and 1.28 (95% CI 1.05–1.56, *P* = 0.01), respectively. Moreover, exclusion of patients with glomerulonephritis (n = 22; 41%) did not affect the results (see Supplementary Table S2); the hazard ratios for renal recovery in the subgroup analysis were in agreement with the main findings of Table 2.

### Evaluation of the Performance of Predictive Models after Addition of Biomarkers in a Conventional Clinical Model

Since higher tubular PRX3 and hemoglobin were independent predictors of AKI recovery, each was added to a conventional clinical model to determine the improvement in the predictive ability of renal function recovery (Table 3). Addition of hemoglobin to the conventional clinical model only significantly increased category-free net reclassification improvement (cfNRI) (63.2%, *P* = 0.02), and addition of tubular PRX3 to the clinical model improved AUROC (0.89, *P* = 0.048), cfNRI (87.4%, *P* < 0.001), and integrated discrimination improvement (IDI) (0.13, *P* = 0.01). Nevertheless, addition of both hemoglobin and tubular PRX3 to the clinical model substantially improved discriminative abilities in terms of AUROC (0.91, *P* = 0.03), cfNRI (88.3%, *P* < 0.001), and IDI (0.19, *P* = 0.001).

## Discussion

To our knowledge, the present study is the first to identify the up-regulation of PRX3, an antioxidant mainly localized in the mitochondria, in the glomeruli and renal tubules of patients with ATN. In comparison with glomerular PRX3, tubular PRX3 was able to predict the recovery of renal function within 6 months after AKI. The expression level of PRX3 in the renal tubules was positively correlated with baseline eGFR but negatively correlated with urinary protein excretion, tubular atrophy, and interstitial fibrosis. High tubular PRX3 expression was independently associated with a higher likelihood of kidney function recovery compared with low tubular PRX3 expression. Furthermore, addition of tubular PRX3 to the conventional clinical model significantly improved the predictive ability of renal function recovery after AKI.

We found that patients with AKI who had high tubular PRX3 expression had less tubular injury and interstitial inflammation. Previous studies have demonstrated the renoprotective role of endogenous antioxidants such as catalase, silent information regulator 1 (Sirt1), Nrf2, and heme oxygenase-1 (HO-1), in AKI^8^,^19^,^20^,^21^. PRXs are a large antioxidant family with limited literature in relation to AKI. Among them, PRX3 is a unique member because it is exclusively localized in the mitochondria and regulates mitochondrial H_2_O_2_ levels^22^. Two studies reported that PRX3 is up-regulated in experimental renal ischemia-reperfusion injury^17^,^18^. Our findings were consistent with those of these studies, demonstrating that renal expression of PRX3 increased in response to AKI. Furthermore, PRX3 depletion has been shown to increase mitochondrial H_2_O_2_ and promote mitochondrial apoptotic signaling^14^. In contrast, overexpression of PRX3 promotes cell survival and prevents tissue remodeling and heart failure^15^,^16^. Our findings are in agreement with the results of the study, suggesting that PRX3, like other endogenous antioxidants such as Sirt1, Nrf2, and HO-1, may have a protective role in renal tubular cells and predict the recovery of renal function after AKI.

Several studies have investigated tubular biomarkers for predicting recovery after AKI. Persistent elevations of urinary neutrophil gelatinase-associated lipocalin (NGAL) and kidney injury molecule-1 are associated with progression of AKI to CKD and may be surrogates for progressive kidney injury after AKI^23^. Furthermore, Srisawat *et al*. reported that plasma NGAL can predict recovery from AKI with an AUROC of 0.74 24. However, addition of plasma NGAL to a clinical model did not improve the prediction of AKI recovery^24^. Two novel urinary AKI biomarkers (tissue inhibitor of metalloproteinase-2 and insulin-like growth factor-binding protein 7) were also reported to predict renal recovery from AKI (AUROC = 0.79)^25^. Similar to previous tubular biomarkers, tubular PRX3 was found to be a fair predictor (AUROC = 0.76) of recovery following AKI. Moreover, addition of tubular PRX3 to the clinical model further improved the prediction of renal recovery. These findings suggest that tubular PRX3 expression may reflect endogenous antioxidant defenses in response to AKI and thus can serve as a suitable biomarker of AKI recovery.

Pre-existing CKD can hamper tubule recovery following AKI and lead to kidney disease progression^26^,^27^. Renal expression of PRX3 including glomerular and tubular PRX3 were evaluated in this study. Both glomerular and tubular PRX3 were upregulated in patients with ATN. However, only tubular PRX3 expression was significantly associated with renal function recovery from ATN. Tubular PRX3 expression level was positively correlated with baseline renal function but negatively correlated with proteinuria and chronic pathological changes (tubular atrophy and interstitial fibrosis). Unlike tubular PRX3, glomerular PRX3 expression level was not significantly correlated with these changes (Supplementary Fig. S2). This indicates that tubular PRX3 may reflect the chronicity of CKD and predict the renal function recovery of patients subjected to AKI. Although clinical variables such as age, sex, diabetes mellitus, need for dialysis treatment, and levels of baseline eGFR, serum albumin, and hemoglobin have been demonstrated to predict renal function recovery following AKI^23^,^28^, addition of tubular PRX3 to the clinical model can further improve recovery prediction.

There are some limitations in our study. First, renal biopsy is usually performed if there is no obvious cause of AKI in clinical practice. Based on the retrospective study design, AKI patients undergoing renal biopsy may have etiologies other than ischemia-reperfusion, sepsis, or nephrotoxicity. Therefore, our results might not represent the relationship between PRX3 and ATN in the whole AKI population. Second, since renal biopsies were not routinely performed for AKI, the relatively small sample size may reduce the statistical power of this retrospective study. To examine whether our sample size was sufficiently large to assess the difference in tubular PRX3 expression between patients with and without recovery from AKI, we conducted a post-hoc power analysis. With a power of 80%, a sample size of 38 would be sufficient to detect the observed difference in tubular PRX3 between the two groups with a two-sided α-error of 0.05. Given the inclusion of 54 patients in this study, the probability of the detected difference with a two-sided α-error of 0.05 was 93%. Moreover, retrospective cohort studies may have lower statistical quality than prospective studies because of potential unmeasured confounders. Third, the relationship between tubular PRX3 expression and renal function or histopathological changes as well as the differential expression between patients with and without recovery may be attributed to renal tubular injury *per se*. Further studies including prospective studies with a larger sample size or basic studies investigating the biological role of PRX3 in renal tubular injury are needed to confirm our results.

In conclusion, high tubular PRX3 expression was associated with a higher probability of renal recovery in patients with ATN. Tubular PRX3 in combination with clinical predictors can help with recovery prediction and risk stratification in patients with AKI undergoing renal biopsy. Our findings suggest that tubular PRX3 might be a novel potential therapeutic target for AKI. Further mechanistic studies are needed to confirm the role of tubular PRX3 in recovery after AKI.

## Materials and Methods

### Study Design and Participants

From January 1, 2000, to December 31, 2004, we retrospectively enrolled 68 adult hospitalized patients with AKI and biopsy-proven ATN at Changhua Christian Hospital (see Supplementary Fig. S3). The enrolled patients were aged ≥18 years and had to meet the Kidney Disease: Improving Global Outcomes criteria for AKI^29^. Exclusion criteria were AKI with prerenal and obstructive etiologies, chronic dialysis patients, kidney transplant recipients, active malignancy, and an insufficient amount of biopsy specimen. Each patient was followed up for 1 year to identify renal recovery from AKI, and 54 patients were finally selected for further assessment. In addition, for the control group, we selected six subjects with normal kidney function and without other significant comorbidities who were undergoing nephrectomy for localized circumscribed renal tumors. Normal renal tissues were obtained from the uninvolved poles of their removed kidneys. All patients provided written informed consent. The study was carried out in strict accordance with guidelines for research involving human subjects developed by the Taiwan Ministry of Health and Welfare and was approved by the Institutional Review Board of Changhua Christian Hospital (approval number 150912).

Renal function tests were performed during follow-up visits until the end of follow-up, complete recovery of eGFR, or death. The primary endpoint was a composite of complete (return to baseline eGFR, within a 10% margin of error) and partial (return to values above baseline eGFR) recoveries of eGFR within 6 months following AKI. Baseline kidney function was determined from the last available stable serum creatinine value within 1 year prior to hospitalization or the lowest inpatient creatinine value prior to kidney failure if outpatient creatinine values were unavailable. Heart failure diagnosis included the diagnoses of congestive or systolic heart failure, diastolic heart failure, or cardiomyopathy based on manual review of medical charts prior to or at the time of AKI. Urinary protein excretion rate (measured by protein-to-creatinine ratio) and demographic data (including sex, age, comorbidities, and medications) were recorded at the time of biopsy. Diabetes mellitus was diagnosed according to the American Diabetes Association criteria^30^. Hypertension was defined according to medical history and/or the use of antihypertensive medication. Among the antihypertensive drugs, angiotensin-converting enzyme inhibitors and/or angiotensin-II receptor blockers were taken by 20% (11/54) of patients.

### Laboratory Methods

Blood hemoglobin, serum levels of creatinine, albumin, total cholesterol, triglyceride, sodium, potassium, and phosphorus, and urine levels of creatinine and protein were measured according to standardized procedures at the Department of Laboratory Medicine, Changhua Christian Hospital. eGFR, calculated by the Chronic Kidney Disease Epidemiology Collaboration formula, was used to evaluate renal function^31^.

### Immunohistochemistry

Immunohistochemistry analyses were performed on formalin-fixed, paraffin-embedded renal tissue sections (4 μm). The sections were placed on coated slides, dewaxed with xylene, and rehydrated in serial dilutions of alcohol, followed by washing with phosphate buffered saline (PBS; pH = 7.2) solution. Endogenous peroxidase activity was blocked by incubation in 3% H_2_O_2_. Antigen retrieval was performed by boiling in citrate buffer (10 mM) for 20 min. After incubation with mouse monoclonal anti-PRX3 antibodies (ab16751; Abcam, Inc., Cambridge, MA, USA) at 1:4000 dilution for 30 min at room temperature, the slides were thoroughly washed three times with PBS. The reaction was visualized using the polymer-based MACH4 DAB Detection Kit (Biocare Medical, CA, USA) according to the manufacturer’s instructions, and the slides were incubated with horseradish peroxidase/Fab polymer conjugate for another 30 min. Finally, peroxidase activity was visualized by incubation with 3, 3′-diaminobenzidine tetrahydrochloride (DAB) as the substrate for 5 min and hematoxylin as the counterstain.

Computer-assisted quantitative analysis was performed as previously described^32^. Briefly, we randomly selected 10 glomeruli and 10 non-overlapping high-power fields (x400) for each renal cortical section and captured images by Olympus Microscope BX51 (Olympus, Tokyo, Japan) equipped with a digital color camera (DP21; Olympus, Tokyo, Japan). The captured images were then analyzed using Image Pro-Plus software (Version 6.0; Media Cybernetics, Silver Spring, MD, USA). QISV was calculated as the integrated optical density divided by total area occupied by the DAB-stained and hematoxylin-stained cells of each slide^32^.

### Histopathology

Formalin-fixed, paraffin-embedded renal biopsy specimens of the 54 patients with ATN and normal renal tissues of the six control subjects were sectioned at 4 μm thickness and stained for histological examination. To determine the severity of tubular injury and percentage of interstitial fibrosis, sections were stained with a periodic acid-Schiff staining kit (Merck Millipore, Billerica, MA, USA) and Masson’s Trichrome Kit (American Master Tech Scientific, CA, USA) according to the manufacturer’s instructions. The percentage of atrophic tubules and fibrosis of cortical interstitium and the severity of tubulointerstitial damage were examined under 10 randomly selected non-overlapping high-power fields (x400). Tubular injury and inflammatory cell infiltration were graded on a 0–4 scale (0, no lesions; 1, lesions involving <25%; 2, lesions involving 25–50%; 3, lesions involving 50–75%; and 4, lesions involving >75% of the cortical parenchyma) as previously described^32^. All sections were examined by a pathologist (T.-C.S.) unaware of the clinical and laboratory data.

### Statistical Analysis

Results are expressed as a percentage, median (interquartile range), or mean ± standard deviation. All variables were tested for normal distribution by the Kolmogorov-Smirnov test. Non-normally distributed variables were either log-transformed (ln) or analyzed by non-parametric statistical tests. Comparisons between the two groups were made by performing Mann-Whitney U test for continuous variables and Pearson’s chi-squared test or Fisher’s exact test for categorical variables. Additionally, ROC curves were constructed to evaluate whether tubular PRX3 expression could serve as a diagnostic biomarker of renal function recovery after AKI. Harrell’s C-index was used to compare the predictive performances of glomerular and tubular PRX3 for discrimination between patients who recovered and did not recover from AKI. Univariate Cox proportional hazards regression analyses were performed to assess the associations between the relevant variables, followed by multivariate analyses with regard to potential confounders. The mortality rate is high in hospitalized patients with AKI^2^. We performed competing-risks regression analyses (Fine-Gray model) based on the Cox proportional hazards model because the risk of kidney function recovery might be confounded by the competing risk of death. The AUROC was calculated with stepwise addition of hemoglobin and PRX3 to a clinical model, including the conventional risk factors (age, sex, hypertension, diabetes mellitus, severity of AKI, urinary protein excretion, and baseline eGFR)^33^, and the change in discriminative ability was calculated to predict renal function recovery within 6 months after AKI. Changes in AUROC, cfNRI, and IDI were estimated to evaluate the incremental predictability of each biomarker when added to the conventional model^34^. In comparison with the model with only conventional risk factors, an increase in AUROC, cfNRI, and IDI was determined in the model with conventional risk factors and new biomarker(s). All statistical analyses were performed using SAS 9.4 (SAS Institute Inc., Cary, NC, USA). *P* values of <0.05 in two-tailed tests were considered statistically significant in all analyses. Post-hoc statistical power analyses were performed using G*Power version 3.1.9.2.

## Additional Information

**How to cite this article**: Wu, C.-L. *et al*. Tubular Peroxiredoxin 3 as a Predictor of Renal Recovery from Acute Tubular Necrosis in Patients with Chronic Kidney Disease. *Sci. Rep.* **7**, 43589; doi: 10.1038/srep43589 (2017).

**Publisher's note:** Springer Nature remains neutral with regard to jurisdictional claims in published maps and institutional affiliations.

## Supplementary Material

Supplementary Materials

## Figures and Tables

**Table 1:** Baseline demographic and laboratory data and histopathology of acute tubular necrosis patients with and without recovery of renal function during the follow-up period.

	Recovery^a^ (n = 33)	Non-recovery (n = 21)	*P* value^b^
***Demographics***			
Age (years)	53.7 ± 20.5	51.5 ± 17.1	0.53^b^
Male (n (%))	22 (66.7)	13 (61.9)	0.72^c^
Diabetes mellitus (n (%))	6 (18.2)	9 (42.9)	0.048^c^
Hypertension (n (%))	5 (15.2)	10 (47.6)	0.01^c^
Heart failure (n (%))	1 (3.0)	2 (9.5)	0.55^d^
Underlying renal disease			0.04^d^
Nil (n (%))	14 (42.4)	3 (14.3)	
Diabetic nephropathy (n (%))	2 (6.1)	4 (19.0)	
Hypertensive nephrosclerosis (n (%))	3 (9.1)	2 (9.5)	
Glomerulonephritis (n (%))	10 (30.3)	12 (57.1)	
Tubulointerstitial disease (n (%))	4 (12.1)	0 (0)	
Severity of AKI			0.50^c^
KDIGO stage 1 (n (%))	6 (18.2)	6 (28.6)	
KDIGO stage 2 or 3 (n (%))	27 (81.8)	15 (71.4)	
***Laboratory data***			
Baseline serum creatinine (mg/dl)	1.0 (0.8–1.4)	2.1 (1.3–3.3)	0.002^b^
Baseline eGFR (CKD-EPI) (ml/min/1.73 m^2^)	69.2 (50.4–92.5)	27.1 (18.3–68.6)	0.006^b^
Urinary PCR (mg/g)	100.0 (56.5–1592.0)	1126.0 (355.1–9957.2)	0.004^b^
Hemoglobin (g/dl)	10.8 ± 2.4	9.0 ± 2.2	0.005^b^
Serum albumin (g/dl)	2.6 ± 0.7	2.7 ± 0.8	0.68^b^
Serum cholesterol (mg/dl)	159.0 (119.0–222.0)	224.0 (157.5–276.5)	0.13^b^
Serum triglyceride (mg/dl)	154.0 ± 101.7	208.6 ± 114.4	0.11^b^
Serum sodium (mmol/l)	135.8 ± 5.9	135.0 ± 5.3	0.53^b^
Serum potassium (mmol/l)	4.0 ± 0.8	4.0 ± 1.1	0.77^b^
Serum phosphorous (mg/dl)	5.1 ± 2.4	6.7 ± 3.4	0.18^b^
***Histopathology***			
Tubular injury score	2.2 ± 1.2	2.6 ± 1.3	0.30^b^
Tubular atrophy (%)	4.3 ± 6.4	16.6 ± 15.4	<0.001^b^
Interstitial inflammation score	0.9 ± 0.5	1.2 ± 0.8	0.21^b^
Interstitial fibrosis (%)	7.8 ± 5.3	11.5 ± 9.4	0.24^b^
***Medications***			
Angiotensin-converting enzyme inhibitor or angiotensin-II receptor blocker (n (%))	5 (17.2)	6 (28.6)	0.49^c^
Immunosuppressants (n (%))	9 (27.3)	7 (33.3)	0.76^c^
Corticosteroid (n (%))	7 (24.1)	7 (33.3)	
Cyclophosphamide (n (%))	1 (3.4)	1 (4.8)	
Azathioprine (n (%))	2 (6.9)	1 (4.8)	
Calcineurin inhibitor (n (%))	1 (3.4)	0 (0)	
Chlorambucil (n (%))	0 (0)	1 (4.8)	

^a^Includes complete recoveries and partial recoveries.

^b^Mann-Whitney U test.

^c^Pearson’s chi-squared test.

^d^Fisher’s exact test.

Data are expressed as n (%) for categorical data and as mean ± standard deviation or median (interquartile range) for continuous data. AKI, acute kidney injury; CKD-EPI, Chronic Kidney Disease Epidemiology Collaboration; eGFR, estimated glomerular filtration rate; KDIGO, Kidney Disease: Improving Global Outcomes; PCR, protein-to-creatinine ratio.

**Table 2:** Cox proportional hazards regression with competing risk of death for renal function recovery within 6 months after acute tubular necrosis.

Parameter variable	Renal function recovery^a^ within 6 months					
	Model 1 adjusted for age and sex		Model 2 adjusted for statistically significant covariates in Table 1		Model 3 adjusted for age, sex and other variables^b^	
	Hazard ratio (95% CI)	*P* value	Hazard ratio (95% CI)	*P* value	Hazard ratio (95% CI)	*P* value
Tubular PRX3 high expression	15.45 (2.18–109.23)	0.006	8.74 (1.22–62.47)	0.03	8.99 (1.13–71.52)	0.04
Hypertension	0.40 (0.14–1.12)	0.08				
Diabetes mellitus	0.42 (0.14–1.24)	0.12				
Tubular atrophy (%)	0.91 (0.82–1.01)	0.08				
Interstitial fibrosis (%)	0.96 (0.90–1.02)	0.15				
Severity of AKI (KDIGO 2, 3 *vs* KDIGO 1)	2.65 (0.81–8.6)	0.11				
Baseline eGFR (10 ml/min/1.73 m^2^)	1.12 (1.00–1.24)	0.04			1.05 (0.93–1.18)	0.41
Urinary protein-to-creatinine ratio (mg/mg)	0.75 (0.62–0.90)	0.002			0.88 (0.75–1.04)	0.14
Hemoglobin (g/dl)	1.33 (1.11–1.61)	0.003	1.26 (1.06–1.5)	0.008	1.28 (1.05–1.56)	0.01
Serum albumin (g/dl)	0.93 (0.58–1.51)	0.78				
Concomitant use of ACEIs or ARBs	0.47 (0.13–1.66)	0.24				
Concomitant use of immunosuppressants	0.64 (0.25–1.63)	0.35				

^a^Includes complete recoveries and partial recoveries.

^b^Model 3 included significant covariates in Model 1.

ACEI, Angiotensin-converting enzyme inhibitor; AKI, acute kidney injury; ARB, angiotensin-II receptor blocker; eGFR, estimated glomerular filtration rate; KDIGO, Kidney Disease: Improving Global Outcomes; PRX3, peroxiredoxin 3.

**Table 3:** Discriminative performances of conventional risk factors and addition of newer biomarkers for prediction of renal function recovery^a^ after acute tubular necrosis.

	AUROC			cfNRI (%)		IDI	
	AUROC (95% CI)	ΔAUROC (95% CI)	*P* value^b^	cfNRI (95% CI)	*P* value^c^	IDI (95% CI)	*P* value^d^
Conventional risk factors only	0.82 (0.70–0.94)	—	Referent	—	Referent	—	Referent
+hemoglobin	0.86 (0.77–0.96)	0.05 (−0.02–0.11)	0.18	63.2 (11.2–115.3)	0.02	0.07 (−0.003–0.14)	0.06
+tubular PRX3 QISV	0.89 (0.81 –0.98)	0.08 (0.001–0.15)	0.048	87.4 (38.3–136.6)	<0.001	0.13 (0.04–0.22)	0.01
+hemoglobin + tubular PRX3 QISV	0.91 (0.84–0.99)	0.10 (0.01–0.18)	0.03	88.3 (39.1–137.5)	<0.001	0.19 (0.09–0.29)	0.001

^a^Risk prediction was assessed by the AUROC, cfNRI and IDI. Each newer marker was stepwise added to the model of conventional risk factors (base model) to assess the AUROC, cfNRI and IDI for predicting recovery of renal function within 6 months. Conventional clinical risk factors included age, sex, hypertension, diabetes, severity of AKI, urinary protein-to-creatinine ratio and baseline estimated glomerular filtration rate.

^b–d^The *P* value for an increase in AUROC, cfNRI and IDI in a model with conventional risk factors and new biomarkers, compared with conventional risk factors alone. AKI, acute kidney injury; AUROC, area under the ROC curve; cfNRI, category-free net reclassification improvement; CI, confidence interval; IDI, integrated discrimination improvement; PRX3, peroxiredoxin 3; QISV, quantitative immunohistochemical staining value; SE, standard error.
