# Supplementary material for: Tubular Peroxiredoxin 3 as a Predictor of Renal Recovery from Acute Tubular Necrosis in Patients with Chronic Kidney Disease
Source: Sci Rep. 2017 Feb 27;7:43589. doi: 10.1038/srep43589 (PMC5378910; doi:10.1038/srep43589)

Tubular Peroxiredoxin 3 as a Predictor of Renal Recovery from Acute Tubular Necrosis in Patients with Chronic Kidney Disease

Chia-Lin Wu, Tzu-Cheng Su, Chia-Chu Chang, Chew-Teng Kor, Chung-Ho Chang, Tao-Hsiang Yang, Ping-Fang Chiu and Der-Cherng Tarng

Supplementary materials

Supplementary Table S1. Harrell's C-index for the predictive performances of glomerular and tubular PRX3 to discriminate between patients with and without recovery from ATN

Supplementary Table S2. Cox proportional hazards regression with competing risk of death for renal function recovery within 6 months after acute tubular necrosis among patients without glomerulonephritis

Supplementary Figure S1. Kaplan-Meier analysis of cumulative incidence of renal function recovery after acute kidney injury among patients with high (solid line) and low (dashed line) tubular peroxiredoxin 3 expression.

Supplementary Figure S2. Relationship of glomerular PRX3 expression with (A) baseline eGFR, (B) proteinuria, (C) tubular atrophy, and (D) interstitial fibrosis.

Supplementary Figure S3. A flow diagram for participants included in the study.

Supplementary Table S1. Harrell's C-index for the predictive performances of glomerular and tubular PRX3 to discriminate between patients with and without recovery from ATN

Biomarkers	C-index (95% CI)	*P* value
Tubular PRX3 QISV	0.67 (0.55–0.79)	0.004
Glomerular PRX3 QISV	0.56 (0.44–0.68)	0.33

ATN, acute tubular necrosis; PRX3, peroxiredoxin 3; QISV, quantitative immunohistochemical staining value.

Supplementary Table S2. Cox proportional hazards regression with competing risk of death for renal function recovery within 6 months after acute tubular necrosis among patients without glomerulonephritis

Parameter variable	Renal function recovery[a] within 6 months					
	Model 1 adjusted for age and sex		Model 2 adjusted for statistically significant covariates in Table 1		Model 3 adjusted for age, sex and other variables[b]	
	Hazard ratio (95% CI)	*P* value	Hazard ratio (95% CI)	*P* value	Hazard ratio (95% CI)	*P* value
Tubular PRX3 high expression	9.27 (1.24–69.28)	0.03	7.97 (1.14–55.71)	0.04	9.39 (1.14–77.14)	0.04
Hypertension	0.47 (0.15–1.51)	0.20				
Diabetes mellitus	0.38 (0.12–1.21)	0.10				
Tubular atrophy (%)	0.95 (0.87–1.04)	0.25				
Interstitial fibrosis (%)	0.98 (0.9–1.07)	0.63				
Severity of AKI						
KDIGO 2, 3 *vs* KDIGO 1	3.06 (0.39–24.22)	0.29				
Baseline eGFR (10 ml/min/1.73m^2)	1.06 (0.95–1.19)	0.31				
Urinary protein-to-creatinine ratio (mg/mg)	0.77 (0.63–0.93)	0.008				
Hemoglobin (g/dl)	1.35 (1.02–1.79)	0.03	1.23 (1.04–1.46)	0.02	1.32 (1.02–1.71)	0.04
Serum albumin (g/dl)	0.76 (0.41–1.38)	0.36				
Concomitant use of ACEIs or ARBs	0.20 (0.02–1.68)	0.14				

Concomitant use of immunosuppressants	0.98 (0.33–2.94)	0.98

ACEI, Angiotensin-converting enzyme inhibitor; AKI, acute kidney injury; ARB, angiotensin-II receptor blocker; eGFR, estimated glomerular filtration rate; KDIGO, Kidney Disease: Improving Global Outcomes; PRX3, peroxiredoxin 3.

[a]Includes complete recoveries and partial recoveries.

[b]Model 3 included significant covariates in Model 1.

Supplementary Figure S1. Kaplan-Meier analysis of cumulative incidence of renal function recovery after acute kidney injury among patients with high (solid line) and low (dashed line) tubular peroxiredoxin 3 expression. tPRX3, tubular peroxiredoxin 3.

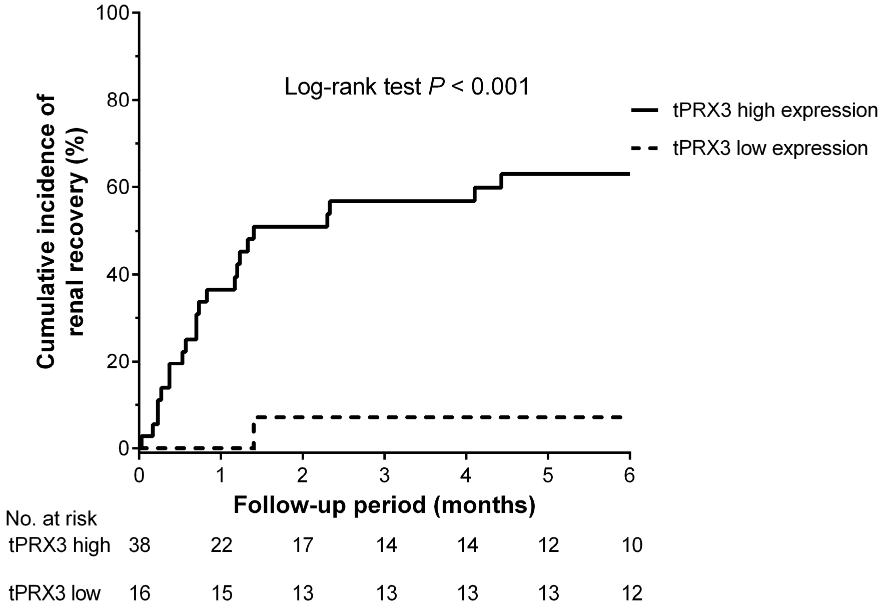

Supplementary Figure S2. Relationship of glomerular PRX3 expression with (A) baseline eGFR, (B) proteinuria, (C) tubular atrophy, and (D) interstitial fibrosis. eGFR, estimated glomerular filtration rate; PCR, protein-to-creatinine ratio; PRX3, peroxiredoxin 3; QISV, quantitative immunohistochemical staining value.

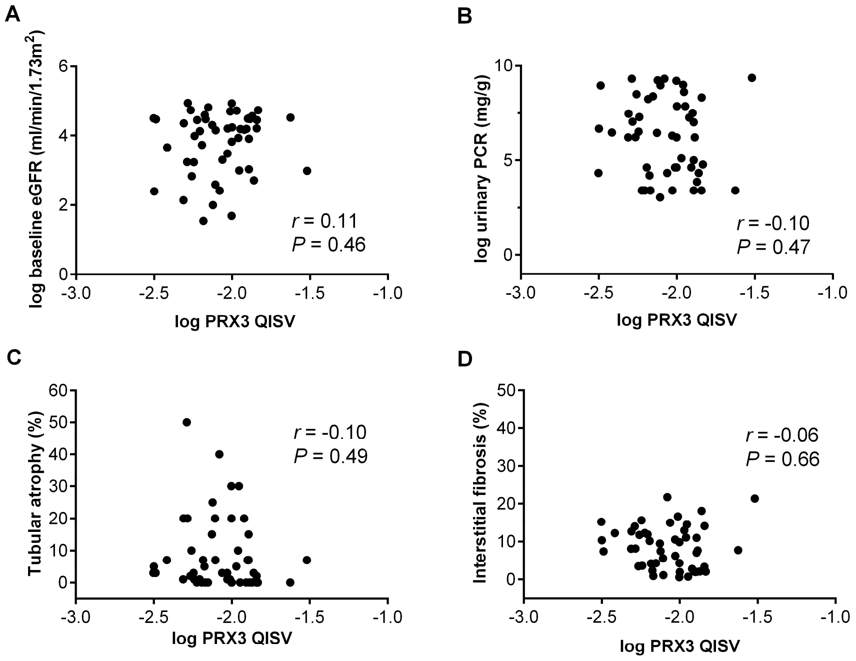

Supplementary Figure S3. A flow diagram for participants included in the study. AKI, acute kidney injury; ATN, acute tubular necrosis.

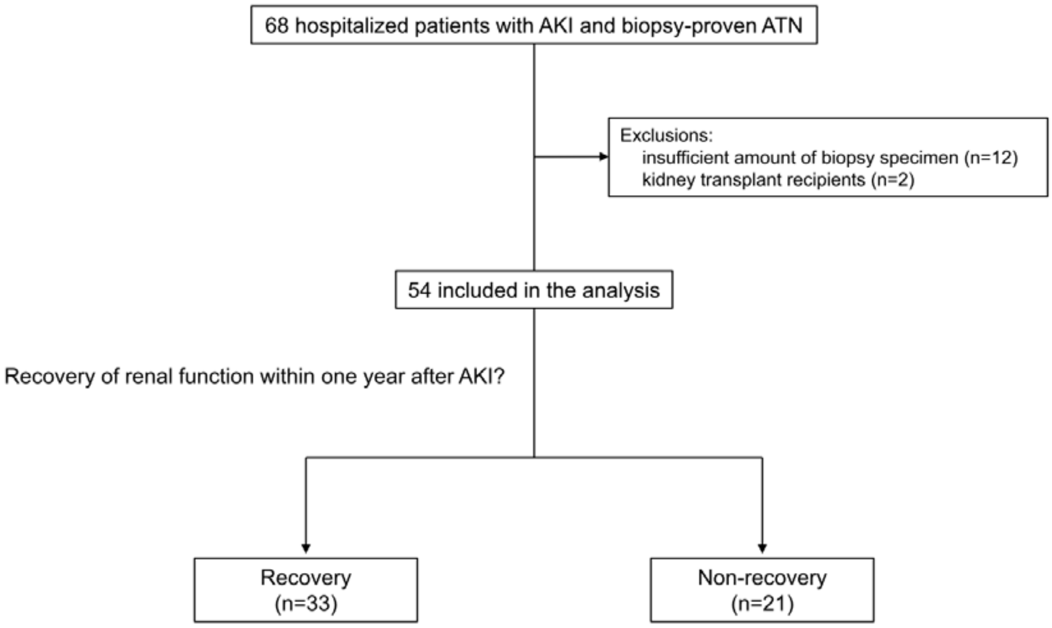

Supplement: Supplementary Materials [file srep43589-s1.pdf]
